# Using Micro-learning on Mobile Applications to Increase Knowledge Retention and Work Performance: A Review of Literature

**DOI:** 10.7759/cureus.5307

**Published:** 2019-08-02

**Authors:** Mrigank S Shail

**Affiliations:** 1 Medicine, Xavier University School of Medicine, Toronto, CAN

**Keywords:** micro-learning, mental fatigue, neuroscience, neuroplasticity, long-term memory, ebbinghaus curve, memory, short term memory, recency effect, primacy effect

## Abstract

Micro-learning is an educational teaching method used to train users on multiple platforms. This article will provide a brief introduction to the concepts of short-term and long-term memory, and explain how micro-learning can be used to increase retention in learners. Micro-lessons can aid in negating the Ebbinghaus forgetting curve and can use reintroduction to keep retention at significantly higher levels. This process also speeds up the learning process overall because students avoid the phenomenon of mental fatigue. The article cites studies suggesting mental fatigue can cause serious cognitive decline in individual performance. By breaking complex courses into manageable smaller lessons, micro-learning preserves the neurotransmitter cascade for steady neurochemical performance. By using mobile devices, students can pause and continue their micro-lessons with ease. The mobile application also gives them the opportunity to continually check on their performance, and adjust their learning accordingly. Micro-learning on mobile devices also keeps engagement levels high because it utilizes different forms of media to keep users captivated.

## Introduction and background

In today's fast-paced world full of distractions and online stimulation, it is very difficult to have a learner sit down in one spot and continually absorb educational material and remain focused on a topic for hours upon hours. Students today find it difficult to keep away from distractions on their mobile devices. Furthermore, learners of today are simultaneously doing multiple other actions instead of purely learning. Many students take online courses because they have a part-time job. Employees lag behind on their compliance training because they are either bombarded with work or cannot spare two or three hours a week to complete the mandatory compliance courses. Both private and public institutes are struggling to keep their employees accredited and compliant. Having employees take long training, learning, or accreditation courses decreases work productivity, cuts into business hours, increases budget spending, and yet does not guarantee full adoption, compliance, or subject matter comprehension. This review aims to explore how micro-learning has evolved from theory to an established educational program. The review will further explore how mobile devices and mobile applications are helping enhance micro-learning for its users. Moreover, the literature will identify the relationships of micro-learning with mobile technology, gaps in micro-learning, and future research into the technology.

What is memory?

The definition of memory is complicated to provide. According to Squire (2004), memory is not a single faculty of the mind but rather is formed of many systems that have various operating principles and several neuroanatomies [1]. Research suggests that memory is formed by creating and linking new neurons together. A specific memory is created by the shape, pattern, and allocation of neurons in different clusters within the brain. Over time, these patterns can be transferred from short-term memory regions (relying more heavily upon the pre-frontal cortex) to long-term memory regions for indefinite storage. The neural system responsible for establishing long-term memory for facts and events consists of several lobes, hippocampus, entorhinal, perirhinal, and parahippocampal cortices [2]. Memories can be relocated to long-term storage by the process of reintroduction and recall. Rehearsing specific memories promote additional neuronal synapses to the neuronal cluster and can change, upgrade, or reinforce the memory. This process of changing memory is known as neuroplasticity, a subject that requires additional research. Neurons primarily transcribe memories in the cortex for the short term. Recalling helps add more neuronal synapses and strengthens memory for long-term storage. Research suggests memory retention can be enhanced if there is an emotional link with the original stimuli, aiding to create connections with the amygdala nuclei (responsible for emotions), laying down stronger neuronal fibers, and cementing the long-term memory [3-5].

What is micro-learning?

Micro-learning is relatively small, focused learning units consisting of condensed learning activities (usually one to 10 minutes), available on multiple devices. The lesson strategies are designed for skill-based training, learning, and education. The short bursts of lessons are also replete with interactive multimedia. It can be used for informal training (with a focus on performance gain) or to teach large, complex material broken down into manageable pieces. Typically designed and delivered in rich media formats, it is a learner-centric approach that provides just-in-time training that is available on multiple devices (Ex: tablets, smartphones, desktops, and laptops) [6]. A 1999 study published in the Journal of Educational Psychology showed that people learn and perform better when they can access short and engaging content at their speed, instead of vast complex information in one session [7]. Hug, 2005, suggested that micro-learning be also known as 'bite-sized' learning because it uses proportioned bite-sized pieces of exercises [8]. Though the concept of micro-learning has been around for a long time, its full potential has only begun to be achieved in recent decades due to the Internet and mobile devices. Equipping learners with material in different formats empowers them to study and later strengthens their knowledge base. As the resources library is online, old content can be updated and new content can be uploaded in real time. With a cornucopia of media formats at its fingertips, micro-learning on mobile applications engages and supports diverse learning styles. Introductory learning material might be long and comprehensive (Ex. clinical paper, course video, company compliance PDF) but micro-lessons would provide succinct summaries for each item in the form of a checklist or a short subject-matter-expert-recorded video, which users can easily manage on the go.

Baumgartner, 2013, further developed the theory of micro-learning and suggested a pathway for success through learning phases: Learning I, Learning II, Learning III, and Learning I+ [9]. In Learning I (absorbing phase), students largely absorb basic knowledge. In Learning II (acquiring phase), students interact with their environment, get active feedback, and create learning experiences. In Learning II (constructing phase), instructors and learners collaborate and create material together to comprehend the subject. On completing these phases, students graduate to a more advanced Learning I+ phase and learn high-level concepts. Similarly, a more social-interactive micro-learning platform was designed by Göschlberger, 2016. In phase I, users build and share content. In phase II, users assess, measure, and enhance the content. In phase III, users tag and accumulate content items. In phase I+, users interact with the content and complete quizzes, which can be retaken to strengthen their comprehension [10].

Technology

Technology has changed substantially in the last decade. Mobile phones have gotten smaller, faster, more powerful, and have more functionality. Suggested by Al Tameemy (2017), these devices are popular among users, which makes them one of the best instruments to be adopted by educational institutions. With over 6 billion subscriptions globally, mobile phones have become an invaluable pathway for that knowledge [11]. Although technology is being hailed as a game-changer for education, there are academics who caution teachers about using technology haphazardly. A 2018 critical review by Pedro et al. concluded that in order for the successful implementation of mobile learning to occur: 1) teachers and students must concentrate more on collaborative-driven practices, 2) teachers should be given adequate training on mobile learning, 3) students should be given sufficient guidance on mobile learning, and 4) parties must acknowledge and readjust to the challenges of distraction and the multitasking behaviour of mobile devices usage [12].

The harbinger of mobile education began with the introduction of the personal computer. In the 1990s, the worldwide web provided unlimited access to a cornucopia of free knowledge and learning material. Combining the Internet and personal computers with Moore's Law, which states that the number of transistors on a microprocessor chip doubles every two years, led to an exponential increase in computer-learning speed and performance in the 2000s [13]. This gave rise to high-speed Internet, mobile devices, social media, mobile applications, and the Internet of all things prevalent in the 2010s.

## Review

Memory curve

Understanding the "forgetting curve" vs the "retention curve."

**Figure 1:**
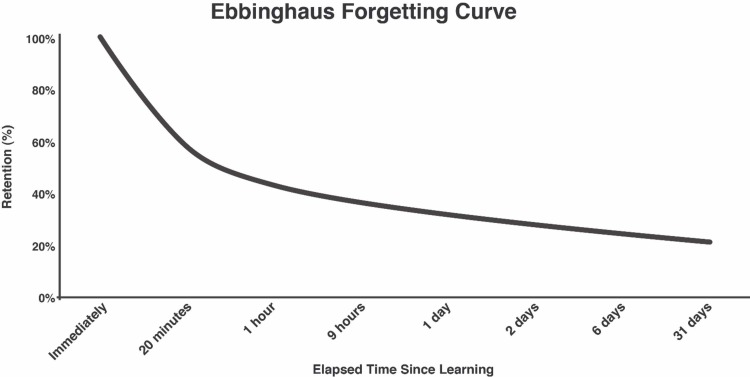
Ebbinghaus Forgetting Curve

Psychologist Hermann Ebbinghaus conducted some of the earliest investigations on memory, recall, and spaced or micro-learning. The 1880 Ebbinghaus curve (Figure 1) or "forgetting curve" theorizes that memory retention decreases over time [14]. It suggests that relevant information is lost through a time when there is no attempt to retain it. A typical "forgetting curve" hypothesizes that participants tend to forget more than 50% of their newly learned material 20 minutes immediately after the lesson ends. Moreover, that learned percentage falls to 40% in nine hours, and then to 24% in 31 days if no revision or repeat learning takes place and all other variables remain constant. Barring any sudden emotional or physical trauma on the participants, they tend to forget the majority of their newly learned material within hours or days. In 2015, Murre and Dros successfully replicated the Ebbinghaus forgetting curve. In their investigation, a subject spent 70 hours learning items at intervals, leading to retention data similar to Ebbinghaus' original study [15].

**Figure 2:**
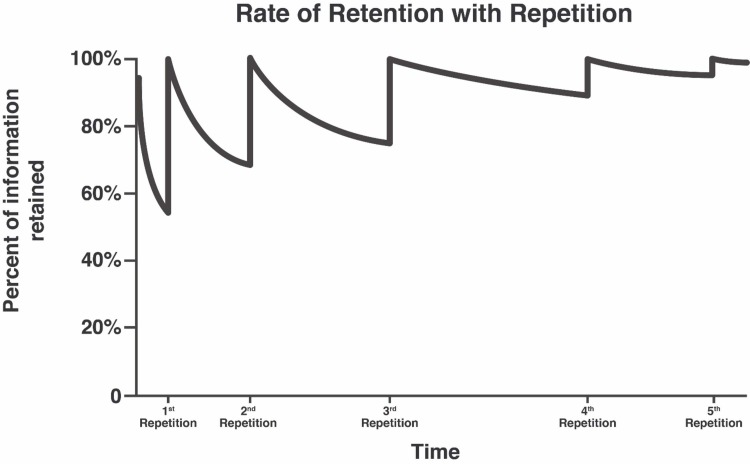
Rate of Retention with Repetition

Reintroducing the lessons in smaller increments will help participants retain knowledge for an extended time. Ample research data supports the claim that memory reactivations can prevent memory impairment or forgetting. Through a process called cellular consolidation, memory undergoes protein synthesis, leading to neuronal changes that alter the memory to long-term memory [16]. This re-introductory process can occur just hours after an introductory learning period and memory traces can be built up. A 2018 MacLeod et al. study states that the reactivations of memory strengthened long-term memory by initiating cellular/synaptic reconsolidation [17]. With the help of personal mobile devices and application, participants can learn at their own pace at any location. With lessons stored on online servers, participants can pause and resume their activities. With the added capability of moving back and forth between lessons, participants can improve their retention percentage by repeating the previously completed lessons in shorter bursts. The process of rehearsing the material creates stronger neural networks connections within the brain and conveys the memory from short-term to long-term. By repeatedly using micro-learning on mobile applications, the retention level can reach to that of early levels or at least plateau off (avoiding a downward curve). According to Kang 2016, hundreds of studies in cognitive and educational psychology have demonstrated that spacing out repeated encounters with the material over time produces superior long-term learning [18]. This repetition technique can migrate learned material into long-term memory more efficiently. Figure 2 shows a hypothetical example of a memory retention curve affected by repetition over time.

Appropriating serial position in micro-learning

Theoretically, by breaking information down into smaller and brief learning moments, micro-learning enables users to focus on one piece of information at a time. It allows for the formulation of intuitive micro-lessons, guiding learners towards a specific learning goal. Educators can also use the design and framework of the course to help users perform better inside micro-learning mobile applications.

Our brains automatically tend to recall the first and last items in a serial list. According to Murphy et al., the position of an item has a significant effect on the attitude towards intention to purchase and affirming a brand or company [19]. This effect even works to create the aura of "first impressions" about people. A pioneering study by Asch found that the order of listing of traits influenced the impression formed from the given set of traits [20]. In the study, a person labeled as “intelligent-industrious-impulsive-critical-stubborn-envious” would have a more favorable first impression than a person labeled as “envious-stubborn-critical-impulsive-industrious-intelligent." All in all, the position of the personality traits determined peoples' attitudes toward that person.

**Figure 3:**
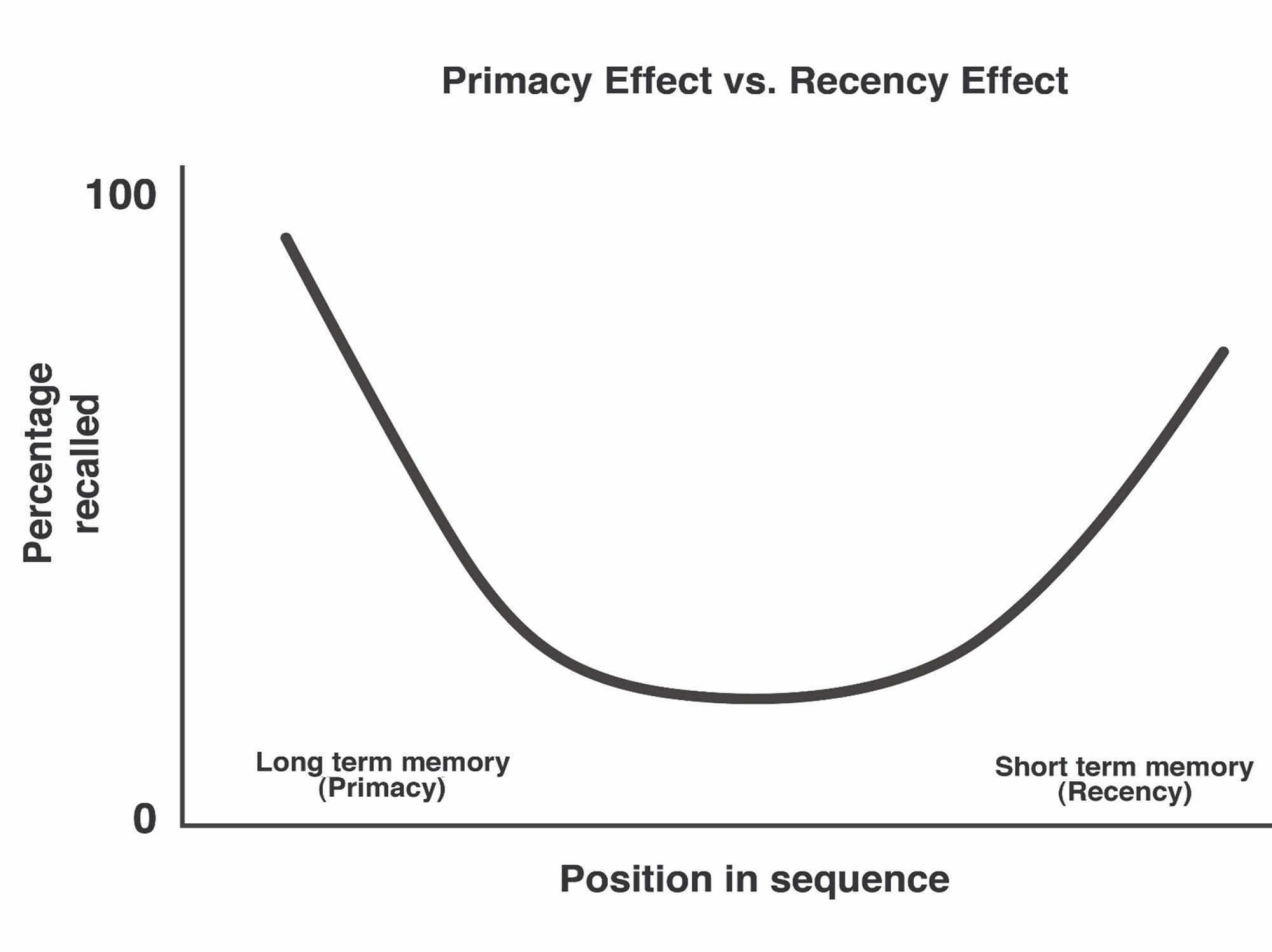
Primacy vs Recency Effect

When remembering a list, items at the beginning and the end are better remembered than items in the middle (Figure 3). Our capacity to recover things at the start of a list is called the Primacy Effect. To retrieve details from the end of a list is called the Recency Effect. The neuroscience evidence of these memory indexing is still under research and review. One theory suggests that as a result of the Primacy Effect, items are stored in long-term memory better when they are presented early on the list. When we see an item at the beginning of the list, we still subconsciously recall that item while going through the rest of the list. Our brain has more time to process the information from the point of the first contact. A Greene et al. study states that, hypothetically, the initial items are better encoded into long-term memory because they have had more opportunity to be rehearsed [21]. Hence, by providing more time to 1) form neuronal synapses and 2) subconsciously recall the items, the Primacy Effect helps migrate memory into long-term memory via neural pathways. Once migrated into long-term regions, memories are linked and processed via the hippocampus - the nuclei responsible for indexing long-term memory for lifetime recall.

A blueprint for strong memory is provided here for clarification:

Direct learning stimuli -> Time to form memory in the short term -> Recall to strengthen connections -> Time to move memory to long-term -> Recall to strengthen long-term connections

In the Recency Effect, items are stored as short-term or "working memory" when they are presented later on the list. These items at the end of the list do not get any time for recall and thus do not get moved into long-term memory storage. However, they are readily available in short-term memory and hence are preferred over other items. Humans use the Recency Effect to hold information for the short term while engaging in other cognitive processes. Without repetition, the memory in the Recency Effect declines with the passage of time and by the presentation of additional information. Recalling short-term memory can transfer them from the short-term memory regions of the brain to the long-term areas of the cortex, indexing them properly via the conduit of the hippocampus. The educator should use the Primacy and Recency Effects when considering how to order items in a list. Moreover, the educator should use the Primacy and Recency Effects when they want learners to remember a specific option or item of importance in the list.

Educators or administrators may use the Primacy and Recency Effects to create micro-learning lessons for students or staff, respectively. Educators may present the most pertinent knowledge concepts at the beginning and end of lists to maximize retention. Administrators may use the technique to update their staff on critical industry compliance regulations.

Micro-learning avoids central fatigue

Micro-learning engages learners by using smaller and specific learning objectives. The short duration of micro-learning content reduces the mental fatigue caused by longer lessons. Students can finish a quick experience, grasp the key concept, and take a break. This method gives time for the learned material to be processed and indexed from short-term to long-term memory. Even though students are not rushed, they tend to finish the entire course faster because they are more immersed. This technique can be accomplished because micro-learning avoids the phenomenon of mental fatigue, also known as central nervous system fatigue or central fatigue.

Mental fatigue can be defined as a person’s inability to efficiently complete cognitive tasks. Per the 2014 study by Ishii et al., it is a potential impairment of cognitive function, and in modern society, it is one of the most significant causes of accidents [22]. Mental fatigue induces a decline in cognitive processes such as planning, response inhibition, executive attention, sustained attention, goal-directed attention, alternating attention, divided attention, and conflict-controlling selective attention [23-24]. At a given moment, there is a finite amount of fuel being provided to the brain in the form of glucose or neurotransmitters. Transmitting synapses and relaying information to different regions of the brain requires constant assembly, uptake, passage, usage, and breakdown of the neurotransmitter. These excitatory and inhibitory compounds are named adrenaline, noradrenaline, serotonin, dopamine, gamma-aminobutyric acid (GABA), acetylcholine, glutamate, and endorphins. Once used, the neurotransmitters need to be reassembled via enzymes, engulfed by voltage-gated calcium channels, and processed by synaptic vesicles. A constantly over-stimulated brain cannot handle the cascade efficiently. According to Kilpatrick and Bressloff, if not given time to rest and recalibrate its neurotransmitter and synaptic vesicle stockpile, the neurons temporary fail to fire and cannot transmit an input signal, leading to synaptic fatigue or short-term synaptic depression [25]. This negates proper long-term memory neuronal connections. Micro-learning uses the conceptual model of neuronal regulation and advocates preventing over stimulations or cognitive exhaustion via multiple time-spaced lessons. With the arrival of smartphones and other mobile devices, micro-learning can be set outside of conventional classrooms with higher adoption rates. Hypothetically, aided by mobile applications, micro-learning can avoid synaptic fatigue and can sustain neuro-chemical regulatory stability. This, in turn, can efficiently maintain the mechanisms of cognitive task performance by avoiding the sensation of mental fatigue.

Mobile devices have enriched and become an intricate part of student life. Technology can be used to engage students outside of the classroom if implemented well. Training and learning can be transformed by the help of self-regulated micro-learning in mobile applications. Students can choose what, when, and how they learn. As a self-access online learning platform, any micro-lesson, in theory, can be taken an infinite number of times. Students can continually self assess their performance - with the option to return to their previous micro-lessons and improve their score through a refresher course. Serial advanced level micro-lessons within the same learning course can then be calibrated as per the user meta-data profile. Micro-learning uses a variable-metric-based synchronized evaluation system. The traditional way to measure effectiveness with exams is not sufficient because part of the information coming from interacting with the mobile application, reintroducing material, and online coaching with teachers will be lost. Caione et al. propose an approach for evaluation based on different variables acquired from the mobile application's social and interactive aspects. The study suggests identifying the critical success factors (CSFs) and key performance indicators (KPIs) of an e-learning method [26].

As a caveat, this review must emphasize that micro-learning does not work for everything. It cannot replace many of today's educational systems. The use of micro-learning via a mobile application can be an augmentation tool to further enhance the experience of learning and training. This paper must also state that there is no set time for a micro-learning lesson, as each brain works at its own pace for learning and cognitive processing. Populations diagnosed with learning disabilities, psychological and psychiatric conditions, neurodegenerative diseases, or neurological medical conditions may not attain the full benefits of micro-learning on mobile applications. Finally, it should be specified that micro-learning must be professionally organized and implemented like a learning syllabus. Online learning platforms such as Khan Academy™, Udemy™, and Coursera™ currently offer courses based on micro-learning. edX™, a massive open online course provider created by Massachusetts Institute of Technology and Harvard University, offers access to thousands of micro-courses from hundreds of partnered institutions worldwide. To achieve the complete advantage of micro-learning on mobile applications, it should follow a neuro-scientific academic guideline in sync with the previously discussed Recency and Primacy Effects juxtaposed with the concepts of short and long-term memory, respectively.

## Conclusions

Micro-learning is a multi-platform educational teaching tool that can be applied to educate a large number of users. This article discusses and explains how micro-learning, juxtaposed with the Primacy and Recency Effects, can facilitate the movement of learned material from short-term to long-term memory. Micro-learning can further be used to increase retention in learners by continually having users rehearse content. The micro-learning process aids students to circumvent the sensation of mental exhaustion. By developing smaller clear exercises and giving needed pauses, the neurotransmitter breakdown-uptake-production cascade can function without being depleted - bypassing severe cognitive decline. Students can pause and continue their micro-lessons anytime, making micro-learning self-paced, which also allows them to return, self-assess, and improve on their previous performance. Users tend to finish lessons faster because micro-learning on mobile devices also keeps engagement levels high because it utilizes different forms of media to keep users captivated. Micro-learning, combined with user metrics, can create an algorithmic platform where lessons plans can be tailored to users' performance and learning curve. Finally, timely reintroductions under the micro-learning produce successful user cognitive knowledge statistics.
